# New achievements in orbital angular momentum beam characterization using a Hartmann wavefront sensor and the Kirkpatrick–Baez active optical system KAOS

**DOI:** 10.1107/S160057752400626X

**Published:** 2024-08-16

**Authors:** Luka Novinec, Matteo Pancaldi, Flavio Capotondi, Giovanni De Ninno, Francesco Guzzi, George Kourousias, Emanuele Pedersoli, Barbara Ressel, Benedikt Rösner, Alberto Simoncig, Marco Zangrando, Michele Manfredda

**Affiliations:** a Elettra Sincrotrone Trieste Strada Statale 14 – km 163,5 in AREA Science Park Basovizza Trieste Italy; b Laboratory of Quantum Optics University of Nova Gorica Nova Gorica Slovenia; c Paul Scherrer Institute Villigen Switzerland; d CNR-IOM – Instituto Officina dei Materiali, Trieste, Italy; DESY, Germany

**Keywords:** tailored photonics beams, orbital angular momentum of light, wavefront sensing, ptychography

## Abstract

Advances in physics have been significantly driven by state-of-the-art technology, and in photonics and X-ray science this calls for the ability to manipulate the characteristics of optical beams. Orbital angular momentum (OAM) beams hold substantial promise in various domains such as ultra-high-capacity optical communication, rotating body detection, optical tweezers, laser processing, super-resolution imaging *etc*. Hence, the advancement of OAM beam-generation technology and the enhancement of its technical proficiency and characterization capabilities are of paramount importance. These endeavours will not only facilitate the use of OAM beams in the aforementioned sectors but also extend the scope of applications in diverse fields related to OAM beams. At the FERMI Free-Electron Laser (Trieste, Italy), OAM beams are generated either by tailoring the emission process on the undulator side or, in most cases, by coupling a spiral zone plate (SZP) in tandem with the refocusing Kirkpatrick–Baez active optic system (KAOS). To provide a robust and reproducible workflow to users, a Hartmann wavefront sensor (WFS) is used for both optics tuning and beam characterization. KAOS is capable of delivering both tightly focused and broad spots, with independent control over vertical and horizontal magnification. This study explores a novel non-conventional ‘near collimation’ operational mode aimed at generating beams with OAM that employs the use of a lithographically manufactured SZP to achieve this goal. The article evaluates the mirror’s performance through Hartmann wavefront sensing, offers a discussion of data analysis methodologies, and provides a quantitative analysis of these results with ptychographic reconstructions.

## Abstract 2

By utilizing the active Kirkpatrick–Baez optical system in collimation mode coupled with a spiral zone plate, orbital angular momentum (OAM) beams have been successfully generated and characterized. The reported findings are relevant for future experiments on OAM structured beams.

## 1. Introduction

In contrast to the visible and infrared spectral regions, where orbital angular momentum (OAM) generation has enabled a wide range of applications, the generation of intense extreme-ultraviolet (EUV) or X-ray vortices remains a challenging endeavour for light source facilities. Essentially, two distinct approaches can be considered: one involves manipulating the emission process to impart OAM inherently to the emitted radiation (native OAM), while the other entails modifying the photon transport through dedicated optical elements to imprint the desired phase profile (optically induced OAM). The first approach typically involves arranging electrons into a helical pattern, achieved either through the use of a seed laser with a suitable transverse phase structure (Ribič *et al.*, 2014 ▸; Hemsing & Marinelli, 2012 ▸) or by the interaction of the electron beam with a laser featuring a Gaussian transverse profile in a helical undulator (Hemsing *et al.*, 2013 ▸; Bahrdt *et al.*, 2013 ▸; Ribič *et al.*, 2017 ▸). The second approach may exploit spiral phase plates (Peele *et al.*, 2002 ▸), computer-generated holograms (Terhalle *et al.*, 2011 ▸) and, more recently, spiral zone plates (SZPs) (Sakdinawat & Liu, 2007 ▸). In particular, the use of SZPs has proved effective for both synchrotron sources (Vila-Comamala *et al.*, 2014 ▸) and free-electron laser (FEL) radiation (Ribič *et al.*, 2017 ▸), where the integrated energy per time unit is orders of magnitude higher.

While both solutions are accessible at FERMI (Trieste, Italy; Ribič *et al.*, 2017 ▸), the generation of native OAM beams remains a desirable but still challenging goal on the machine side, especially when uniform intensity distributions, tuneable topological charges and sign are required. In addition, the divergence of OAM beams increases with the topological charge (Padgett, 2017 ▸), potentially inducing a clipping on the beam at high topological charges, thus limiting the efficiency of the photon transport system within specific spectral ranges. For these reasons, optically induced OAM beams are generally preferred in experiments thus far, as they are simpler and faster to produce. However, the level of practicality and flexibility in using these masks ultimately compares with the optics available on the beamline. For instance, let us examine the varying behaviour of a zone plate (ZP) with a given focal length when positioned downstream of an ellipsoidal mirror compared with a Kirkpatrick–Baez (KB) system, both initially producing a stigmatic spot. With the ellipsoidal mirror, even after the introduction of the ZP (*e.g.* slightly out of focus), the novel spot remains stigmatic. In contrast, with the KB system, which inherently exhibits two distinct focal lengths in the tangential and sagittal directions, the resulting spot from the ZP will inevitably be astigmatic. With two different focal planes, the ZP illumination is not homogeneous, resulting in asymmetry of the OAM beam intensity. The issue is circumvented if the KB system features an active curvature control, as in the case of the DiProI endstation at FERMI. Here, for example, the vertical and horizontal exit arms are *q*_V_ = 1.75 m and *q*_H_ = 1.2 m, respectively. When coupled to a SZP of focal length *f*_SZP_ = 164.7 mm, they would produce an astigmatic difference (*i.e.* the separation between the foci) of ∼5.7 mm, which is far from being acceptable for most experiments. Over the past few years, we have developed a novel approach to tune the performance of the Kirkpatrick–Baez active optic system (KAOS) well beyond its original optical design [which aimed to provide a tightly focused spot on the sample stage (Raimondi *et al.*, 2019 ▸)], which is to compensate for the natural divergence of the source and deliver a near-collimated beam. Similar work has been reported by Goto *et al.* (2016 ▸) on two-stage active KB mirrors, compared with a single stage in our experiment. The optical surface is tuned to achieve a radius of curvature matching exit arms ranging from tens to thousands of metres, so reducing the astigmatic difference from ∼5.7 mm down to between 200 µm and 10 µm, depending on the specific case. Such capability has been already exploited in four studies (Jal *et al.*, 2019 ▸; Rösner *et al.*, 2020 ▸; Fanciulli *et al.*, 2022 ▸; Pancaldi *et al.*, 2024 ▸) conducted on the DiProI beamline.

In this work, we will enter into the details of the process, discussing the metrological aspects and the relevance of wavefront sensing. We will generate OAM beams by illuminating a set of SZPs with variable integer topological charge ℓ = 0, ±1, ±2, ±3. During the process, we will first assess the wavefront quality during the collimation process, aiding in the optimization of the optical surfaces. Second, we will evaluate the wavefront quality of the resulting OAM beam related to the metrological properties of the optical surfaces. These aspects will be addressed in Sections 3 and 4, respectively. Special attention will be given to the effects of surface error defects of the mirror on the resulting wavefront and on the accurate estimation of the OAM. Lastly, since this study was conceived to enable the first proof-of-principle ptychographic OAM imaging measurements at the FERMI FEL, Section 5 will present a comparison between the phase of OAM beams as detected by a Hartmann wavefront sensor and via ptychography, using the same setup as that of Pancaldi *et al.* (2024 ▸).

## 2. Experimental layout

**Figure 1:**
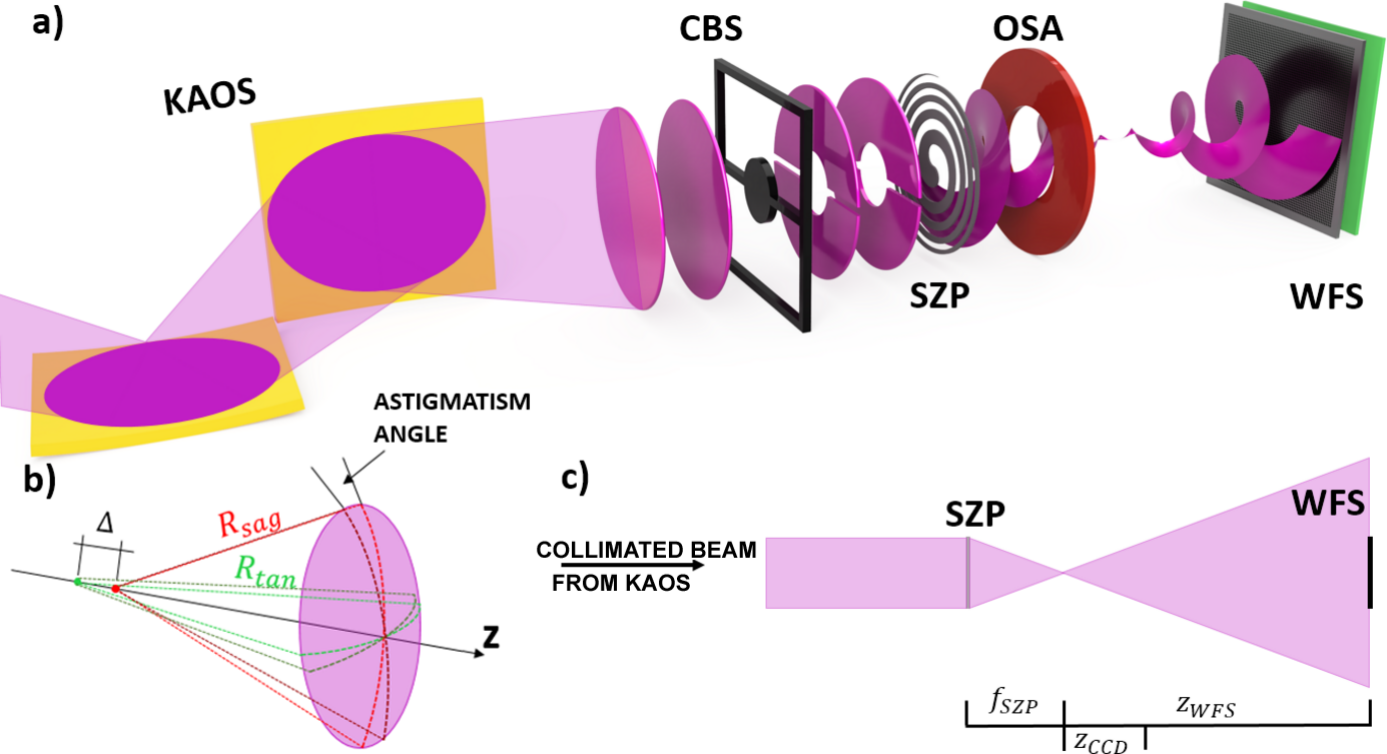
(*a*) The layout of the experimental setup on the DiProI beamline. With KAOS tuned to beam collimation, the SZP with a focal distance *f*_SZP_ generates a focused OAM beam in front of the OSA, which selectively permits light from the first diffraction order to reach the sample but blocks other diffraction orders. Positioned in front of the SZP optics, a CBS obstructs the transmitted direct beam. The Hartmann wavefront sensor is mounted at a distance *z*_WFS_ downstream from the SZP focal plane. Numerical values are: *f*_SZP_ = 164.7 mm, *z*_WFS_ = 1620 mm and *z*_CCD_ = 140 mm. (*b*) The relation between the sagittal and tangential focusing radii. In a perfectly stigmatic system, the distance between two source points is 0 (Δ = 0). (*c*) A sketch depicting the focused beam with the detection area (not to scale).

The experiment was performed on the DiProI beamline (Capotondi *et al.*, 2013 ▸) of the FERMI FEL source (Allaria *et al.*, 2012 ▸) in parallel with the ptychographic OAM imaging experiment (Pancaldi *et al.*, 2024 ▸), aimed at testing the role of structured light in enhancing spatial resolution [Fig. 1 ▸(*a*)]. KAOS is used to illuminate a set of SZPs, while an order sorting aperture (OSA) stops light of higher diffraction orders, letting only the first diffraction order that carries the OAM propagate. By choosing a particular ZP, the topological charge can be chosen, whereas all of the SZPs are designed to have the same focal length (*D* = 1.92 mm, *d*_*r*_ = 1642 nm). At a wavelength of 18.9 nm (horizontal polarization), the resulting nominal focal distance is *f*_SZP_ = 164.7 mm. With this concept, switching between different topological charges can be achieved by the use of a piezo-motorized stage that simply positions the desired ZP in the beam. The precision of the lateral positioning is not important as long as the ZP is fully illuminated, which was carefully monitored. A central beam stop (CBS) blocks the unfocused direct beam. Finally, a Hartmann wavefront sensor (WFS) is mounted at a distance *z*_WFS_ = 1620 mm in the diverging beam downstream of the focal position [Fig. 1 ▸(*c*)]. For comparison with the ptychography reconstruction described in Section 5, a Siemens star test plate (Horstmeyer *et al.*, 2016 ▸) is inserted near the focal plane delivered by the SZP, and an EUV-sensitive in-vacuum Princeton MTE2048 CCD camera collects the scattered radiation at a distance *z*_CCD_ = 140 mm. In this layout, ptychography and Hartmann wavefront sensing are mutually exclusive.

Wavefront detection is performed using a commercial Hartmann wavefront sensor (HASO EUV by Imagine Optic, Orsay, France). It features a square-pinhole mask array (72×72 pinholes) mounted 20 mm in front of an EUV-sensitive CCD. The displacement of the diffraction spots with respect to their geometric projection provides a direct measurement of the wavefront gradient (slopes), which is numerically integrated (Southwell, 1980 ▸) to obtain the wavefront (λ/100 resolution) (Varkentina, 2020 ▸).

## 3. Beam collimation

KAOS comprises two optically coated fused silica plane mirrors (400 mm × 40 mm × 10 mm each), which can be curved independently to an (almost) elliptical shape using mechanical benders. In its typical operational modes, KAOS can focus radiation either precisely at the nominal sample plane (focusing mode), resulting in sharply focused spots (r.m.s. lateral size 1–12 µm, dependent on the wavelength), or operate out of focus (around a few hundred millimetres), generating broader spots of the order of hundreds of micrometres (shaping mode). Metrological details about the typical optical surfaces’ figure error that can be achieved by means of this system, and considerations of the quality of the focal spot obtained, supported by numerical simulations and measurements, can be found in the work of Raimondi *et al.* (2019 ▸) and Simoncig *et al.* (2021 ▸) and in the references of Manfredda *et al.* (2022 ▸). Collimation, however, is considered as an additional advanced operation mode as in this case. For this mode, which was originally not foreseen, the KB curvature is relaxed much beyond the original design range, to compensate for the natural divergence of the source, thus producing a nearly plane wavefront. This is possible by accepting some compromises in shape error, due to the non-ideal bending of the optical surfaces outside the design parameters of the KB system.

### 3.1. Wavefront curvature

**Figure 2:**
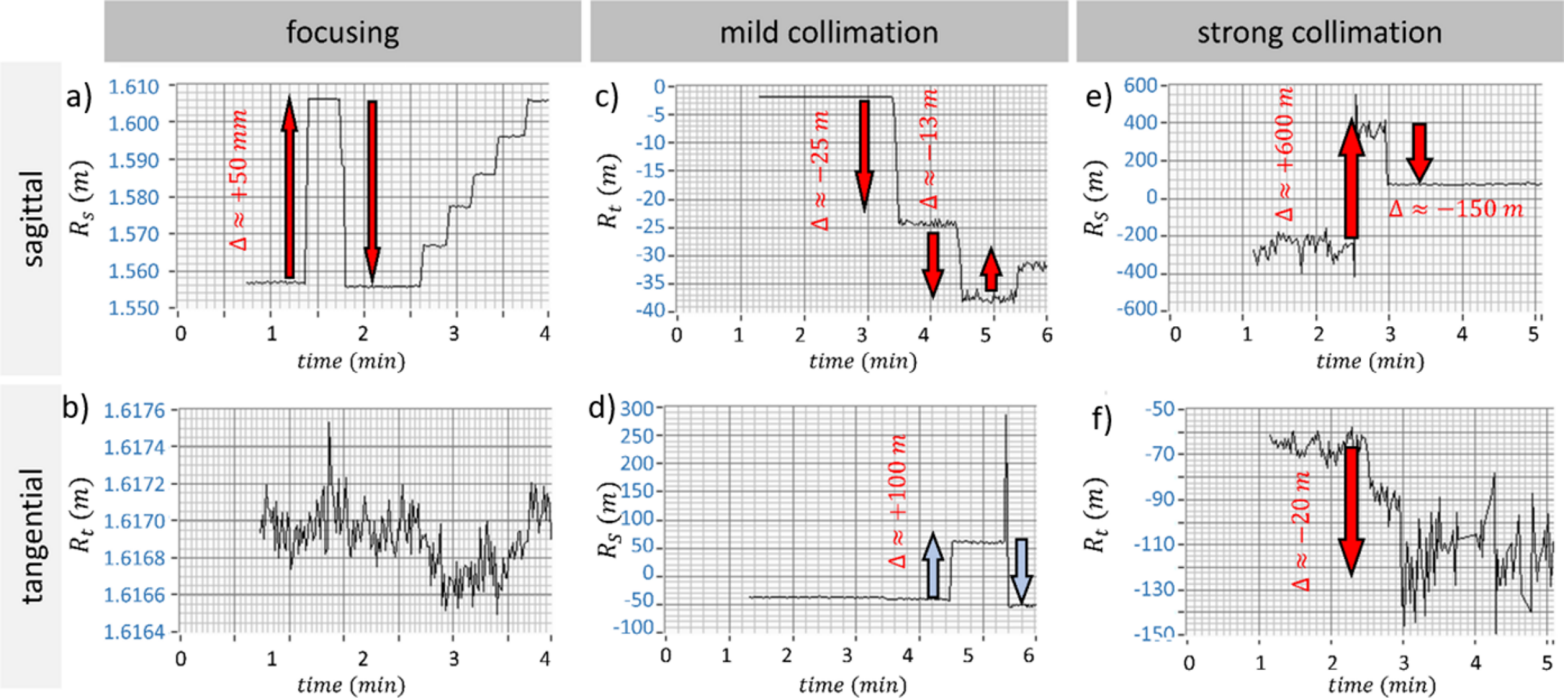
Plots of *R*_s_ and *R*_t_ for varying degrees of collimation. (*a*) The sagittal radius undergoes a variation Δ*R*_s_ ≃ 50 mm (either in a single-step or a multi-step motion), while (*b*) the tangential radius remains unaffected. This adjustment is reversible and allows for the removal of astigmatism by equalizing *R*_s_ and *R*_t_. In (*c*) and (*d*) *R*_s_ remains constant as *R*_t_ is varied by an amount Δ*R*_t_ = −25 m. However, *R*_s_ exhibits a consistent variation (Δ*R*_s_ ≃ 100 m) when *R*_t_ is further varied by an amount Δ*R*_t_ ≃ −13 m (from *R*_t_ ≃ −25 m to *R*_t_ = −38 m). A similar situation is observed for very large wavefront radii in panels (*e*) and (*f*), where the controlled variation in the sagittal direction (Δ*R*_s_ ≃ 600 m) induces a variation in the tangential direction (Δ*R*_t_ ≃ −20 m). In addition, a subsequent step back in the sagittal direction (Δ*R*_s_ ≃ −150 m) does not correspond to a similar change in the tangential direction. This indicates that the process is not entirely reversible.

The relaxation of the optical surfaces is achieved by measuring and controlling the tangential and sagittal curvature radii of the emerging wavefront, *R*_t_ and *R*_s_, respectively, and measured by means of the Hartmann sensor. Within an ideal aberration-free KB system, *R*_t_ and *R*_s_ are oriented as the KB system axes [Fig. 1 ▸(*b*)] and can be independently tuned by adjusting the vertical (V) and horizontal (H) mirrors, respectively. This is shown in Fig. 2 ▸, which illustrates how the sagittal and tangential radii change as a function of the curvature of the vertical KB substrate. Here, we identify three different qualitative behaviours in terms of how the tuning of one radius affects the second one, as a function of the collimation length. Specifically, in the focusing mode [Figs. 2 ▸(*a*) and 2 ▸(*b*)], *R*_s_ varies without affecting *R*_t_ in a reproducible way. Note that both *R*_s_ and *R*_t_ are close to the wavefront focal plane distance *z*_WFS_. Instead, in the ‘mild-collimation’ scenario, where the KB mirrors deliver curvature radii of the order of tens of metres [Figs. 2 ▸(*c*) and 2 ▸(*d*)], the behaviour shifts: beyond a certain threshold, a variation in *R*_t_ also induces a variation in *R*_s_ and with a limited reproducibility. The effect is even more pronounced in the case of ‘strong’ collimation [Figs. 2 ▸(*e*) and 2 ▸(*f*)], where the detected radii are of the order of hundreds of metres. Moreover, within this range, variations in tangential and sagittal radii become non-reproducible. This implies that, while in focusing mode small changes in the actuator position result in small variations in wavefront radius (1 µm at the actuator results in wavefront curvature variation between 1 mm and 2 mm), in collimation mode even a small variation in the actuator position can lead to substantial changes in the wavefront curvature itself (1 µm at the actuator results in wavefront curvature variation of the order of tens of metres). Additional details are provided in the caption of Fig. 2 ▸. This is based on the simple fact that the bending radius of the mirrors is inversely proportional to the actuator position, whereas small changes are more pronounced for large bending radii.

We identify the main source for the cross-talk between *R*_t_ and *R*_s_ in the residual twist affecting the optical surface that is not adequately compensated by KAOS’s mechanical anti-twist system. Consequently, a 45° astigmatism term is introduced into the wavefront, which cannot be rectified through the other available degrees of freedom (pitch and roll).

### 3.2. Residual wavefront

**Figure 3:**
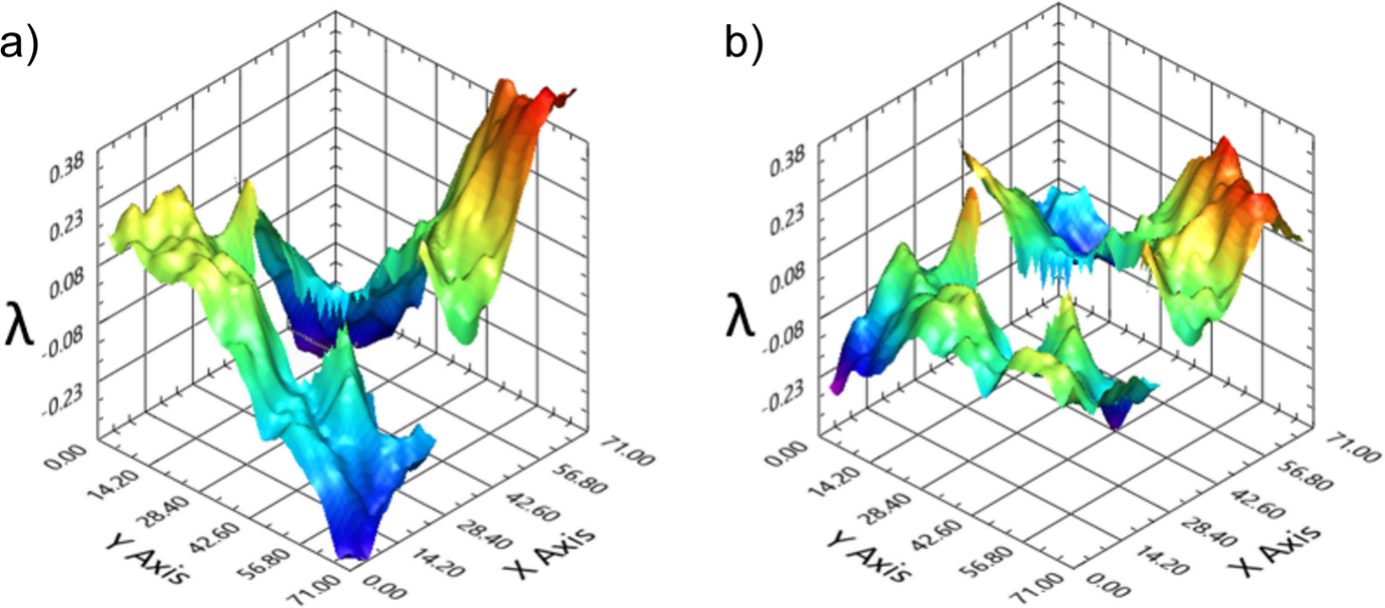
(*a*) The residual wavefront of ℓ = 0, with a notable astigmatism contribution. (*b*) The same data, with numerical subtraction of astigmatism.

The signature of 45° astigmatism is visible in Fig. 3 ▸(*a*) which shows the residual wavefront (with respect to a sphere) focused by a Fresnel ZP with the same optical parameters (with ℓ = 0). Notably, it exhibits a characteristic saddle shape, with a peak-to-valley (PV) error (relative to the spherical wave) of approximately λ and r.m.s. wavefront deformation of approximately 0.23λ (4.3 nm). In Fig. 3 ▸(*b*) the same data are presented after numerical subtraction of astigmatism, yielding to a wavefront which is mostly due to the figure error defects of the optical surfaces. Indeed, the resulting r.m.s. is around 0.1λ, which is approximately twice as large as the r.m.s. value obtained with a similar wavelength operating KAOS in best focusing mode (r.m.s. = 0.05λ). As such a value can also be a little bit larger (up to 0.1λ), we conclude that the major effect in the wavefront due to KB collimation is still dominated by the 45° astigmatism, due to the twist of the optical surfaces. Further details about the distinct contributions affecting the wavefront will be presented in the next section.

### 3.3. Choosing the work point

**Figure 4:**
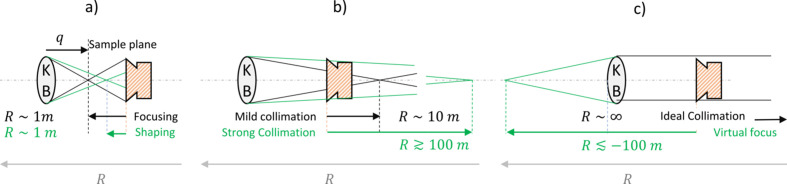
A conceptual sketch of the progressive KB relaxation. (*a*) The focus lies either in the nominal plane, at a distance *q* from the KB centre (focusing mode), or a little bit away from it (tens of millimetres). The detected *R* is typically around the one metre mark. (*b*) By progressively relaxing the KB curvature, the focus shifts away, falling into the so-called ‘mild collimation’ (*R* of the order of a few metres) and ‘strong collimation’ (*R* in the order of tens or hundreds of metres) regimes. (*c*) A further KB relaxation leads first to ideal collimation (*R* → ∞) and then to a diverging beam (produced by a change in the substrate curvature). Experimentally, the collimation regime corresponds to positive-to-negative fluctuations of *R* around ±10^4^ m, which conceptually corresponds to fluctuations around ±∞.

In Section 3.1 we presented two collimation modes: mild and strong. Generally, when a ZP with focal length *f*_ZP_ is placed downstream of the KB mirrors, it focuses the radiation at a distance 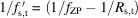, so to minimize the astigmatic difference between the two focusing radii, 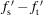, large values of |*R*_*s*,*t*_| are needed, and thus strong collimation is theoretically the best candidate to minimize astigmatism. However, in this range the wavefront curvature radii exhibit erratic behaviour, making curvature control challenging, which consequently impacts the SZP’s focusing distance. For this reason, mild collimation is sometimes preferred, depending on the experimental activities. Fig. 4 ▸ illustrates a sketch depicting the change in wavefront curvature.

Until now, we have used SZPs in two main applications that require different degrees of astigmatism control: a study based on projection imaging of the OAM beam after sample interactions, and an experiment based on the reconstruction of an image on the speckle diffraction pattern. In projection imaging experiments, utilizing either Fresnel ZPs (Jal *et al.*, 2019 ▸; Rösner *et al.*, 2020 ▸) or SZPs (Fanciulli *et al.*, 2022 ▸), the beam is typically collimated once during beamline tuning without further manipulation during beam time, making the use of strong (though erratic) collimation feasible.

In imaging experiments, however, we have experienced that further adjustments of the KAOS optical curvature may be desirable, such as for enhanced astigmatism correction or for addressing small drifts in machine configuration. Hence mild collimation, with reduced erratic behaviour of the wavefront curvature radii during KAOS fine tuning, is preferred. This is the case of interest here, which presents the same condition as the work presented by Pancaldi *et al.* (2024 ▸).

## 4. OAM diagnostics

### 4.1. Wavefront composition

We tuned KAOS in the mild collimation mode in such a way as to minimize the difference between the horizontal and vertical beam dimensions. After illuminating the SZP with an optimized beam, the goal is to evaluate the quality of the resulting wavefront and to test the capability of the Hartmann wavefront sensor in assessing the beam topological charge in the presence of an aberrated beam.

The total 2D wavefront can be written as
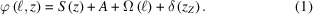
In polar coordinates (ρ, ϑ) the terms are as follows: *S*(ρ, ϑ) = *k*ρ^2^/2*z*_*Z*_ is the wavefront of the (diverging) spherical wave (with *k* the wavenumber and *z*_*Z*_ ≡ *z*_WFS_ the distance between the SZP focal plane and the observation plane), Ω_ρ,ϑ_ = ℓϑ is the wavefront of the OAM beam with (integer) topological charge ℓ, 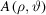 = 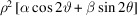 is the summation of vertical and oblique astigmatisms, and δ(ρ, ϑ) is a coarse-grain noise-like contribution due to the figure error of the optical surfaces. To limit the effect of the jitter affecting the beam pointing, every wavefront φ is the result of averaging over *N* = 60 shots. The average r.m.s. fluctuation of the detected wavefront is of the order of 0.5 nm (∼λ/38). The variables (ρ, ϑ) take discrete values mapped over (*x*_*i*_, *y*_*k*_), where (*i*, *k*) iterate over the image pixel indexes. Both subscripts and the dependence on *z* (typically constant) will be inferred when not explicitly stated.

### 4.2. Wavefront differential analysis

An effective way to remove the unwanted wavefront contributions *S*, *A* and δ to isolate the OAM term Ω(ℓ) involves using the property Ω(−ℓ) = −Ω(ℓ). We thus define the semi-difference wavefront as
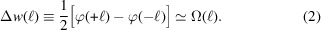
Such an approach allows us to remove the contributions from the reference sphere, the astigmatism and the surface defects without the need to know their analytical expressions (in contrast to the results depicted in Fig. 3 ▸ for ℓ = 0, where a numerical fit of *A* was used).

Since measurements at ±ℓ are taken over different SZPs, the very good equality due to the precision of the manufacturing process accommodates negligible differences which might arise from different plates. We can also define the semi-sum wavefront,
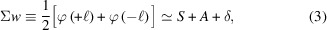
from which we can extract the joint contribution of astigmatism and figure error δ + *A* ≃ Σ*w* − *S*_fit_, where *S*_fit_ is the numerical fit in the slope domain. In principle, δ alone can also be obtained as δ ≃ Σ*w* − *S*_fit_ − *A*_fit_. However, fitting the astigmatic term may give less accurate results.

**Figure 5:**
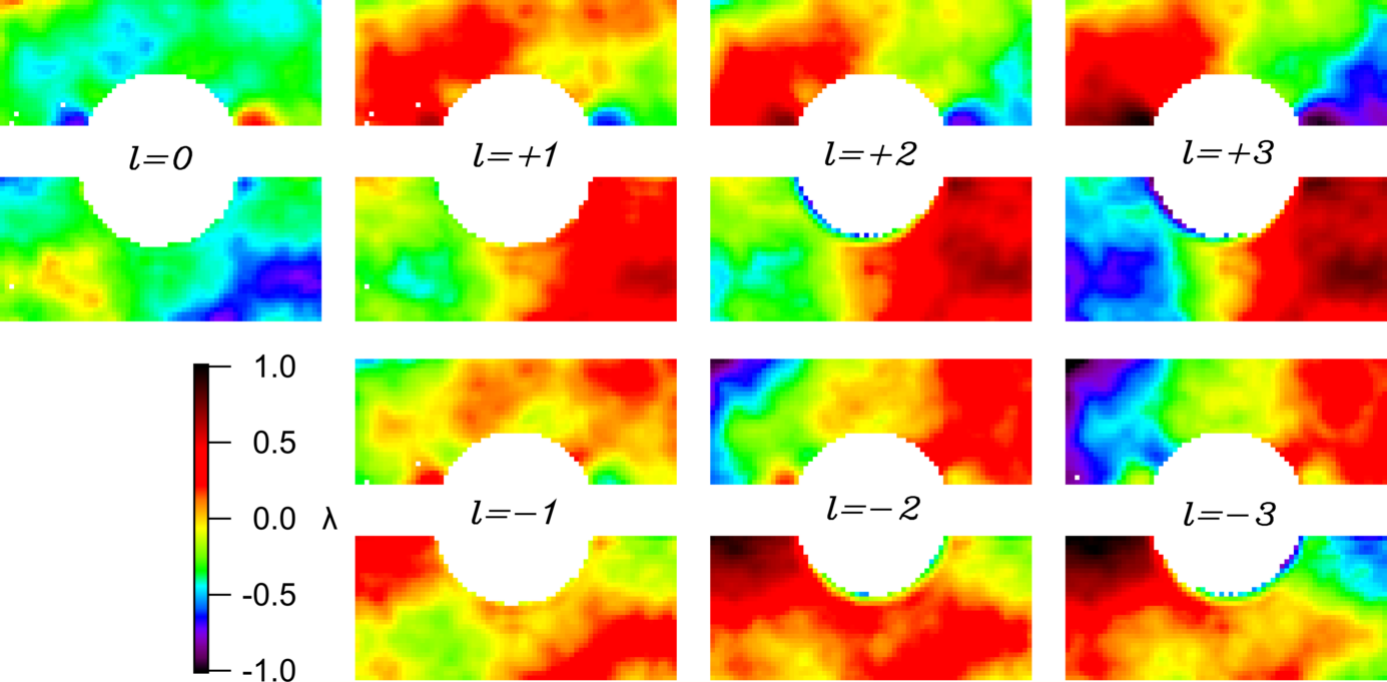
Total (averaged) wavefronts φ(ℓ) for ℓ values from −3 to 3.

**Figure 6:**
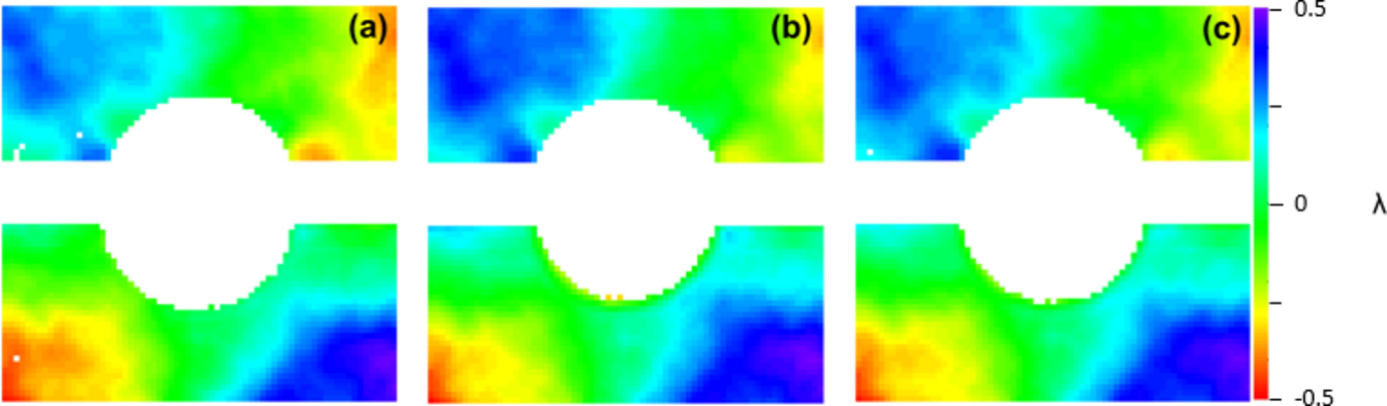
Wavefront contribution of the astigmatism *A* and figure error δ, Σ*w*(ℓ) − *S*_fit_ ≃ (*A* + δ). The three panels, (*a*) |ℓ| = 1, (*b*) |ℓ| = 2 and (*c*) |ℓ| = 3, are almost identical [with the same standard deviation (s.d.) of 0.22λ], as expected.

**Figure 7:**
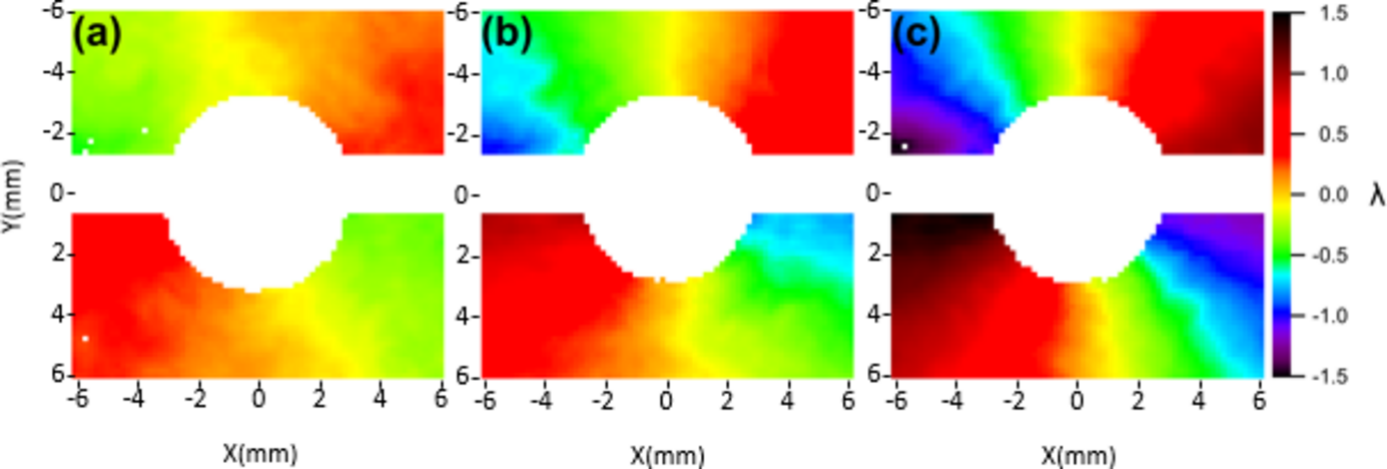
Average differential wavefronts Δ*w*(ℓ) for (*a*) ℓ = 1, (*b*) ℓ = 2 and (*c*) ℓ = 3.

The averaged raw residual wavefronts are computed as follows from φ(ℓ) − *S*_fit_ (see Fig. 5 ▸). As mentioned in the previous section, here the wavefront appearance is mostly dominated by astigmatism. A progressive increase in PV is noticeable from lower to higher |ℓ| values due to the OAM term. In equation (3) one can note that δ is independent of ℓ and we expect δ_ℓ=1_ ≃ δ_ℓ=2_ ≃ δ_ℓ=3_. Fig. 6 ▸ shows the joint contributions of astigmatism (*A*) and figure error (δ) computed for different values of ℓ. The wavefronts display no substantial variation as a function of ℓ, supporting the correctness of equation (1). Note that both the astigmatic and figure error contributions feature a PV value of the order of 1λ, which makes it comparable with the contribution of low-order OAMs. This makes background subtraction via equation (2) advisable for a correct OAM diagnosis. In Fig. 7 ▸ we present the averaged differential wavefronts Δ*w*(ℓ) for ℓ = ±1, ±2, ±3. Here, the appearance of the phase vortex is immediately visible at a glance.

**Figure 8:**
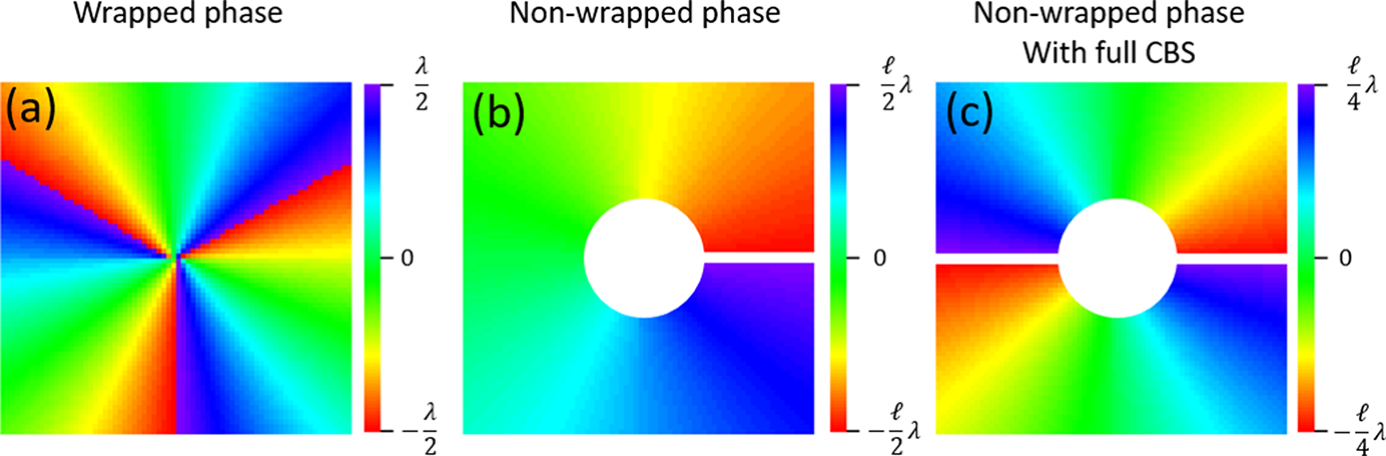
Different ways of representing the OAM phase distribution for the case of ℓ = 3. The phase is represented over three ranges with a total extension equal to (*a*) λ, (*b*) ℓλ, (*c*) ℓ/2λ. Due to CBS, our results are presented as a non-wrapped phase (*c*), which is discontinuous over 2π.

Among the two possible ways of representing phase, either wrapping it over a single period λ or allowing it to span multiple wavelengths, we have chosen the latter. This choice aligns better with the numerical process followed to integrate the displacement field to obtain the scalar wavefront field. In addition, the presence of the CBS necessitates performing the numerical integration over the upper and lower domains separately. This separation makes it impossible to adjust the phases into a single continuous representation without assumptions about their mutual offset. Consequently, the phase values approximately span the range [−ℓλ/4, ℓλ/4] twice (over the two halves), rather than the range [−ℓλ/2, ℓλ/2] once (over the full circle). A summary of three possible representations is shown in Fig. 8 ▸. As a concluding remark, we observe that Ribič *et al.* (2017 ▸) used a plane mirror to illuminate the SZP, so with negligible figure error, thus permitting a simpler OAM detection without the need for a differential approach.

### 4.3. Least-squares fit

**Figure 9:**
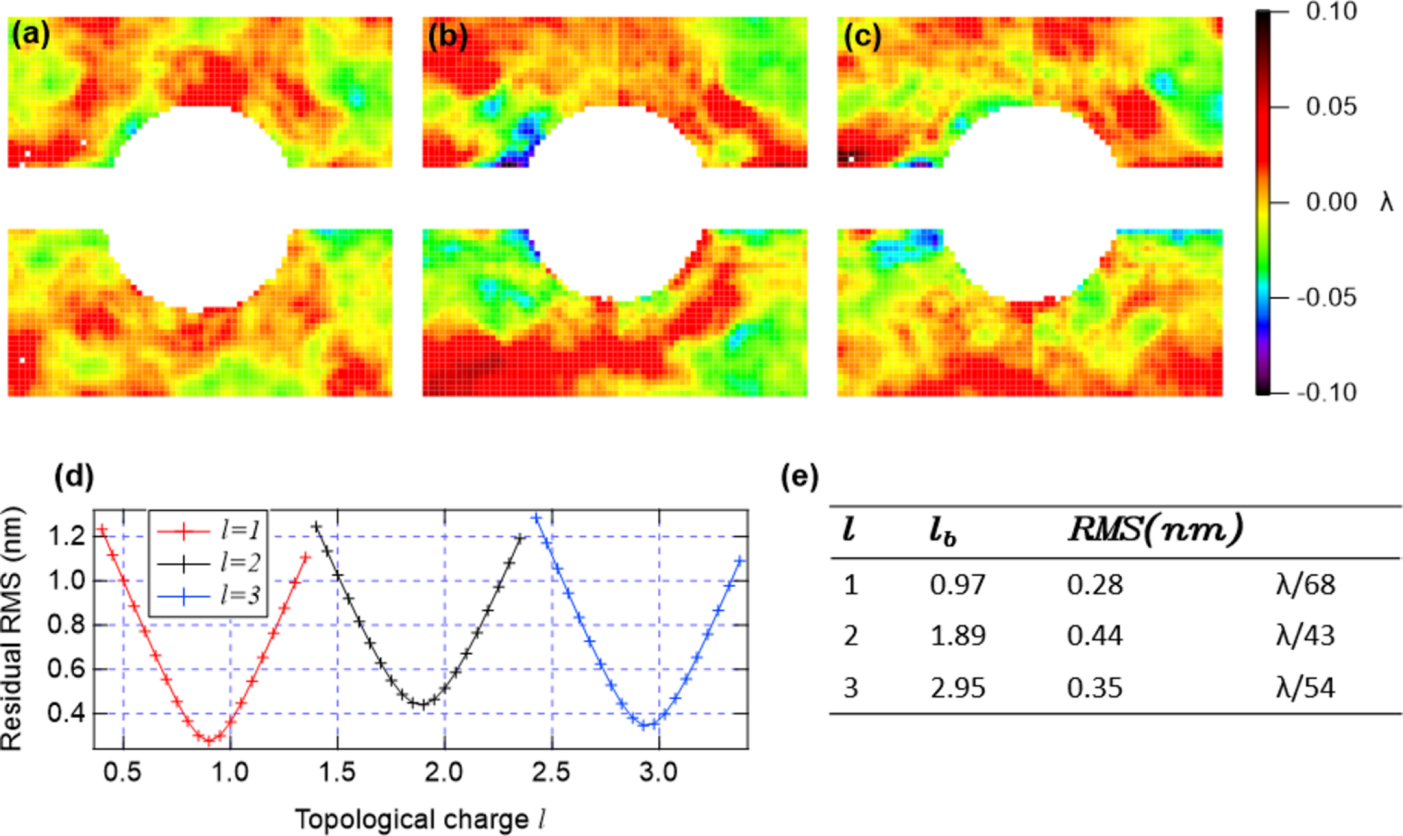
The best fit residual was obtained for (*a*) ℓ = 1, (*b*) ℓ = 2 and (*c*) ℓ = 3. (*d*) A plot of the fitting results with valleys representing a minimum in the r.m.s., calculated over the 2D best fit residual image. (*e*) Fitting results show low r.m.s. values with ℓ_b_ within ∼5% error of the expected integer values ℓ.

To give a quantitative evaluation of the topological charge, we minimize the sum of squared residuals *E*(ℓ′) = 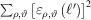, where ɛ(ℓ′) = Δ*w*(ℓ) − ℓ′ϑ is the residual wavefront relative to analytical expression. The summation iterates over the image pixels. The best value ℓ ≡ ℓ_b_ is computed as the mean of the best values obtained on the two domains separately. The results are reported in Fig. 9 ▸. Panels (*a*), (*b*) and (*c*) display the best value residual wavefronts ɛ(ℓ_b_) which exhibit a noise-like pattern. This indicates good OAM purity. Panel (*d*) shows the r.m.s. of *E*(ℓ′) as a function of the topological charge where the minimum value corresponds to ℓ_b_. Those values are reported in panel (*e*). The determined values ℓ_b_ align with the expected values within a ∼5% margin of error.

### 4.4. Non-differential analysis

It is reasonable to wonder what accuracy we can expect in cases where the differential analysis is not accessible and the contribution due to the figure error δ cannot be removed. The results obtained by fitting the quantity φ(ℓ) − *S*_fit_ − *A*_fit_ ≃ Ω(ℓ) + δ are reported in Table 1 ▸ for all ℓ values within the experimental set. In the absence of differential analysis, the resulting ℓ_b_ falls within a 60% margin of error, which is a much higher error than with differential analysis.

Moreover, the r.m.s. value calculated over the best fit residual 2D image is approximately an order of magnitude higher than with differential analysis. This confirms that, for an accurate OAM assessment in this framework, removing the contributions of astigmatism and figure error is better achieved by differential analysis than numerical subtraction.

## 5. Comparison with ptychography

Ptychography (Faulkner & Rodenburg, 2004 ▸) is a lensless imaging technique where the complex field of a scattering object is reconstructed from a set of diffraction patterns obtained through raster scanning. As a result, both the complex field at the sample plane and the complex illumination function (CIF) are obtained (Rodenburg & Maiden, 2019 ▸) as a superposition of non-coherent modes. While Pancaldi *et al.* (2024 ▸) investigated the effects on the sample field resolution, here the CIF is considered as an alternative wavefront diagnostics, similar to the work of Sala *et al.* (2019 ▸).

**Figure 10:**
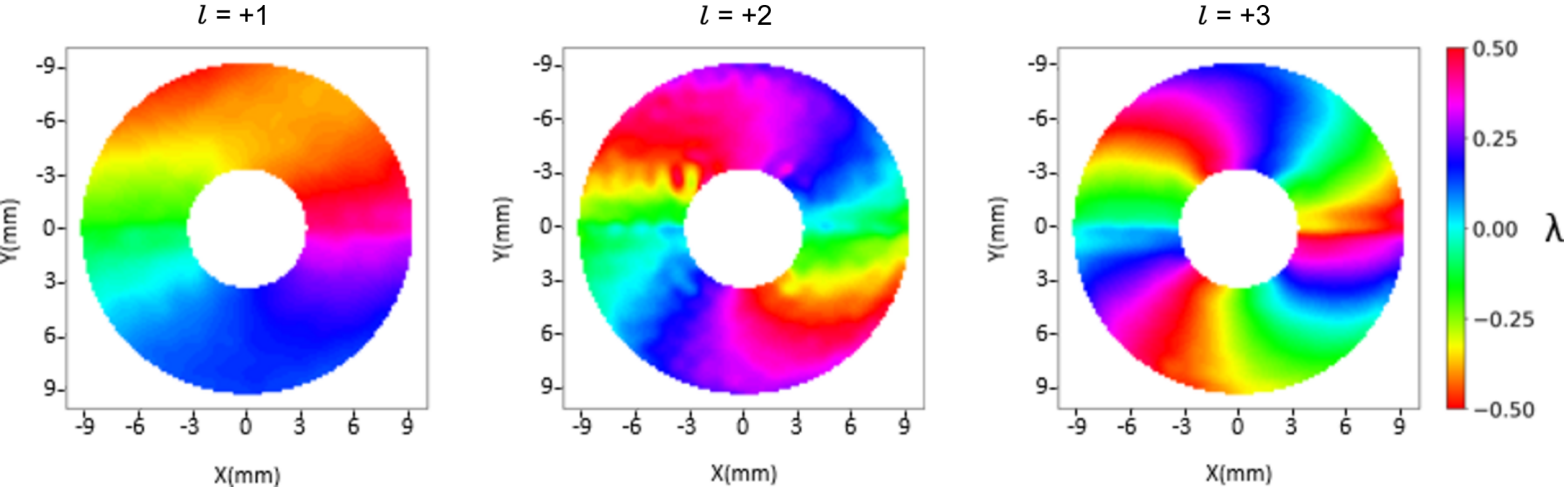
The phase of the complex illumination function (CIF) obtained via ptychographic reconstruction.

A 100 nm-thick HSQ Siemens star structure (Horstmeyer *et al.*, 2016 ▸) patterned on top of a 200 nm-thick silicon membrane is inserted in the focal plane of the SZP. For each ℓ value of the SZP, the Siemens star is scanned in a 7×7 grid while being illuminated by a single FEL shot. The diffracted intensity is recorded by an in-vacuum Princeton CCD camera, placed at a distance of 140 mm from the sample. The phase of the reconstructed CIF is then numerically propagated to the same plane as the Hartmann sensor, as shown in Fig. 10 ▸ (only the higher mode is displayed, containing more than 80% of the total power).

When comparing Fig. 7 ▸ and Fig. 10 ▸ two differences stand out. First, due to the spherical term *S*(*z*) in equation (1), the phase is radially constant over curved lines, while in Fig. 7 ▸, where the spherical contribution is subtracted, the phase is constant along straight lines. Second, and most notably, the effect of the topological charge is evident in ptychography (as emphasized by the colour map) without the differential analysis required for the Hartmann wavefront sensor. Indeed, since the phases in Fig. 10 ▸ correspond to single values of ℓ, they are expected to resemble those in Fig. 5 ▸ more than Fig. 7 ▸. This means that astigmatism and figure error do not significantly influence the CIF phase.

**Figure 11:**
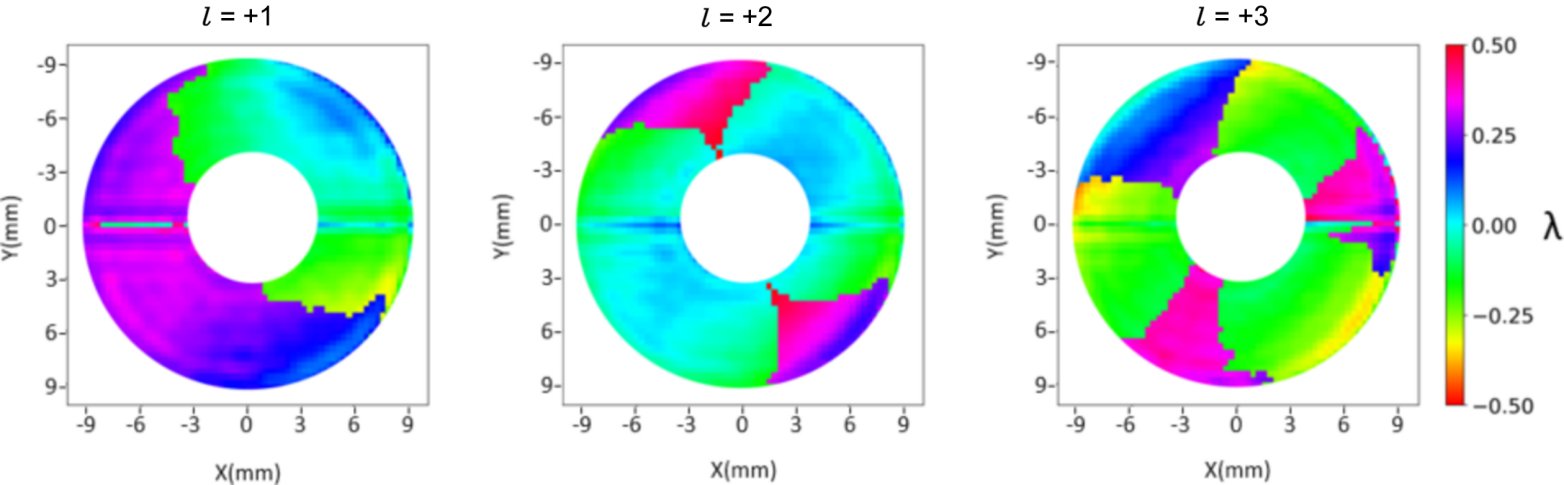
Values of 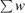 computed for the CIF phases of different ℓ values.

**Figure 12:**
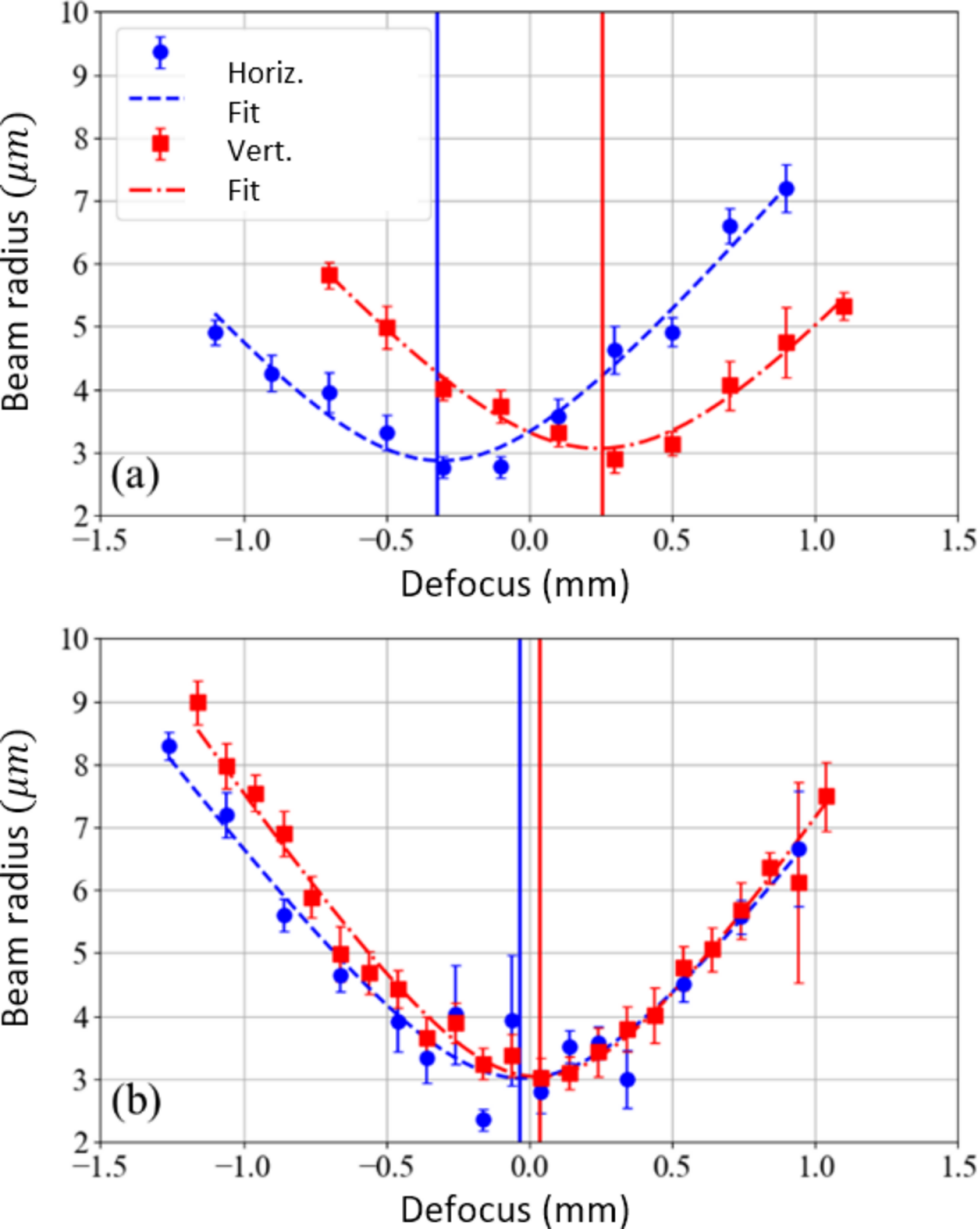
The spot size of the reconstructed beam as a function of defocus, computed for vertical and horizontal cuts. (*a*) Before and (*b*) after tuning the KAOS astigmatism angle. The separation between minima is reduced from ∼1000 µm to ∼70 µm, corresponding to mirror *R*_s_ ≃ 16 m and *R*_t_ ≃ 30 m.

In fact, in Fig. 11 ▸, the quantity Σ*w* computed for the phases of the CIF (intending to extract *A* + δ) appears different from Fig. 6 ▸: it shows a strong non-physically justified dependence on ℓ and the astigmatism is not visible. However, as extensively discussed by Pancaldi *et al.* (2024 ▸), ptychography successfully reconstructs the astigmatic spot and it shows good sensitivity to variations in KAOS optical surface bending. For example, Fig. 12 ▸ displays the horizontal and vertical sizes of the reconstructed spot as a function of the defocus distance, before [panel (*a*)] and after [panel (*b*)] tuning the KAOS astigmatism angle.

These seemingly contradictory behaviours can be reconciled by assuming that astigmatism and figure errors primarily affect the CIF’s amplitude, not its phase. An astigmatic beam illuminating an SZP creates a distinct half-cut spot in intensity [supplementary information in the paper by Pancaldi *et al.* (2024 ▸)]. Our hypothesis is that such an evident intensity feature is incorrectly attributed to the CIF’s amplitude, possibly involving higher-order illuminating probes. Notably, this effect is not inherently tied to the magnitude of the wavefront disturbance. Astigmatism displays a peak-to-valley variation of the order of λ, comparable to OAM with ℓ = 1. Similarly, there is no clear link to the spatial scale of the disturbance: neither astigmatism (spanning the entire azimuth 2π) nor figure error contributions (with features developing only over some fraction of the beam size) appear in the CIF phase. Instead, the OAM, covering the entire azimuth, dominates the CIF phase. Further investigation of the tuning of ptychographic reconstruction, in order to reproduce a CIF phase more closely resembling the real one, could be beneficial for future applications.

## 6. Conclusions

This paper reports the results of generating OAM beams at EUV wavelengths by coupling the KB system (KAOS) with *ad hoc* manufactured SZPs. The experiment was conducted at the FERMI FEL (λ = 18.9 nm), where we employed KAOS in a non-standard mode to produce a nearly collimated beam, enhancing endstation capabilities while introducing minor oblique astigmatic contributions (PV ≃ 1λ). Using a Hartmann wavefront sensor, we characterized the KAOS relaxation and assessed the topological charge of the resulting OAM beams. Our analysis focused on minimizing astigmatism effects and ensuring system operability by evaluating wavefront behaviour, including curvature radii adjustments.

In addition to astigmatism, we detected a residual wavefront error of approximately 0.1λ (r.m.s. value) due to mirror figure errors, comparable with that observed when KAOS is used in its ordinary focusing mode. This underscores astigmatism as the primary cause of wavefront deterioration. We mitigated astigmatism and figure error contributions through differential wavefront analysis, achieving accurate topological charge determination with approximately 5% error. Non-differential analysis yielded less reliable results.

Wavefronts detected by the Hartmann sensor were compared with ptychographic reconstruction images. Differences in wavefront visualization methods between ptychography and the Hartmann sensor were also discussed. Specifically, ptychographic reconstructions revealed the CIF phase containing OAM contributions but lacked astigmatism and figure error information.

The wavefront analysis methodology, the algorithms employed and our assessment of the impact of mirror surface errors on wavefront detection provide valuable insights for applications involving FEL-generated OAM beams or other structured light beams directly produced by FEL sources. With six KAOS systems deployed across FERMI and FLASH EUV FEL facilities, our findings hold significant potential for practical applications in this field.

## Figures and Tables

**Table 1:** The results obtained by performing a least-squares fit on non-differentiated wavefront images

ℓ	−3	−2	−1	0	1	2	3
ℓ_b_	−2.5	−1.5	−0.3	0.6	1.6	2.3	3.5
R.m.s. (nm)	3.26	3.61	2.35	2.36	2.46	2.74	2.52
	λ/6	λ/5	λ/8	λ/8	λ/8	λ/7	λ/7
